# Institutional Housekeeping: Answers to Correspondents

**Published:** 1911-11-11

**Authors:** 


### Institutional Housekeeping.

ANSWERS TO CORRESPONDENTS.

"Lady Guardian" asks: What would be a sufficient housekeeping allowance for an isolation hospital under the care of a man and his wife? At present there is one nurse and one patient, the latter on milk diet, but I should like to know how much ought to be allowed also during convalescence.

∴ Much depends on the arrangement made with the caretakers originally. It is usual to pay an all-round salary in such cases and let them board themselves, as these little hospitals are frequently empty. If this is done, and if the caretaker is trustworthy, it would be wiser not to disturb the arrangement but to pay in 12s. a week extra for the nurse's board and let them provide it. It might be provided for less than this sum, but it must be remembered that during the time when the hospital is occupied the caretakers will themselves expect to live a little more generously, and by paying an adequate sum they will be enabled to do this. If malt liquor is provided it should be ordered and paid for separately. The caretaker must receive exact instructions as to general character of the meals to be provided, and should be instructed to consult with the nurse about her tastes. The milk bill for the patient should be kept entirely distinct. The average quantity of milk required on milk diet is three pints a day, costing 6d. But beef-tea is a very common adjunct to so-called milk diet, and this, if home-made, will cost at least 8d. a pint. A good concentrated preparation, such as Brand's, Liebig's, Bovril, or Oxo, is to be preferred to the home-made article under these circumstances, and should be provided as required on the nurse's requisition. In convalescence much trouble and probably expense would be saved by paying an inclusive sum for the patient's board, similar to that suggested for the nurse. The meals would be ordered by the nurse, and any extras sanctioned by the doctor would be paid for separately. But for 24s. a week a very satisfactory table, with poultry and fish as required, could be provided. There is no economy in stinting the allowance, as any attempt to do this invariably results in a strong pull against economy in other directions.—Ed. T.H.

